# Research on Optical Metrology for Complex Optical Surfaces with Focal Plane Wavefront Sensing

**DOI:** 10.3390/mi14061142

**Published:** 2023-05-28

**Authors:** Xinxue Ma, Jianli Wang, Bin Wang, Xinyue Liu, Yuqiang Chen

**Affiliations:** Changchun Institute of Optics, Fine Mechanics and Physics, Chinese Academy of Sciences, Changchun 130033, China

**Keywords:** complex surface 1, surface measurement 2, optical metrology 3, wavefront sensing 4

## Abstract

Complex optical elements have the advantages of improving image quality and optical performance and expanding the field of view. Therefore, it is widely used in X-ray scientific devices, adaptive optical elements, high-energy laser systems, and other fields and is a hot research direction in precision optics. Especially for precision machining, there is a greater need for high-precision testing technology. However, how to measure complex surfaces efficiently and accurately is still an important research topic in optical metrology technology. In order to verify the ability of optical metrology for complex optical surfaces with wavefront sensing based on image information of the focal plane, some experiment platforms in different types of optical surfaces were set up. In order to validate the feasibility and validity of wavefront-sensing technology based on image information of focal planes, a large number of repetitive experiments were carried out. The measurement results with wavefront sensing based on image information of the focal plane were compared with the measurement results with the ZYGO interferometer. The experimental results demonstrate that good agreement is obtained among the error distribution, PV value, and RMS value of the ZYGO interferometer, which shows the feasibility and validity of wavefront sensing based on image information of focal plane technology in optical metrology for the complex optical surface.

## 1. Introduction

Because the use of complex optical elements can make the spatial layout of the optical system more flexible, that is, increasing the degree of freedom of the system design while reducing the number of system elements, more and more complex optical elements are used in the optical system of high-accuracy optical elements. However, the increase in complex optical elements and the increasingly high requirements for their surface accuracy has brought great challenges to optical processing and testing. Especially for precision machining, there is a greater need for high-precision testing technology. The optical testing technology of complex surfaces has become a research hotspot for scholars [1,2,3]. Compared with traditional spherical and aspherical surfaces, complex surfaces challenge the concept of traditional optical systems and have many advantages in optical performance, space, and weight limitations. The use of complex surfaces allows greater freedom and flexibility in the design and manufacture of optical elements, which lets complex surfaces have greater freedom in aberration correction and control of light direction [4]; this can eliminate various aberrations of optical systems and simplify the optical system, which can make its structure more compact and have higher optical performance [5]. Complex surfaces usually have large phase gradients; thus, optical testing of complex surfaces is extremely challenging [6,7].

Optical testing methods for complex surfaces can be divided into two types: the point–line measurement method, such as the coordinate measurement machine (CMM), and the profilometer measurement method [8,9]. The point–line measurement method needs to scan point by point or line by line, which is slow and inefficient. For example, the CMM method uses point-by-point scanning to measure, which is slow and cannot obtain the full field shape data of the measured element at one time. The swing arm contour scanning method is also faced with problems of low measurement efficiency and errors in the overall surface shape stitching process. At present, it can only measure freeform surfaces of off-axis aspheric types. The research on measuring the high degree of freedom surfaces with complex shapes, large local gradient changes, and difficult mathematical expression of surface shape has not been reported. The other method is plane measurement. In recent years, more and more attention has been paid to plane measurement methods, which mainly include interferometric testing and structured light 3D measurement. The measurement accuracy of interferometry is high, including sub-aperture stitching technology [10,11], computer-generated hologram (CGH) [12], and fringe reflection method [13]. Among the most popular methods, interferometry is a standard measurement method for high-precision polished optical surfaces and is currently the most accurate optical testing method. However, aspheric optical elements are very difficult to test due to their different internal curvature radii. CGH is the commonly used null interferometry in interferometric testing [14], but it also faces several problems: the mode of one-to-one compensation measurement causes its poor measurement versatility, so the testing cost is high. For curved surface components with large gradients, CGH, as a compensator, needs to achieve the output of a large gradient wavefront through a diffraction structure with high density, so the groove density of CGH is limited by the current level of microstructure processing technology. The non-null interferometric method is the sub-aperture stitching method, but it also faces several problems: excessive sub-aperture division will greatly reduce the measurement efficiency and will also bring difficulties to the surface shape stitching, resulting in the decline of the overall shape stitching accuracy. It has high requirements for mechanical adjustment, high cost, and requires a lot of testing time, so it is not widely used in testing complex optical surfaces. 

In recent years, wavefront sensing [15] could be divided into pupil plane wavefront sensing and focal plane wavefront sensing according to the exit pupil position of the optical system [16]. Additionally, the wavefront sensing technology based on image information of the focal plane, which is also called focal plane wavefront sensing (FPWS), has attracted more and more attention from scholars. FPWS is in the image plane of the imaging optical system position and often does not need to add the auxiliary optical components, which capture the multi-frame short-exposure image by given the defocus aberration, the solver that obtains the wavefront phase information of the optical system and can use Zernike polynomials fitting the individual aberrations. Compared with interferometer and other testing methods, FPWS can dynamically test optical components and systems. Phase retrieval (PR) is one of the FPWS methods that pays more attention to the algorithm design and mainly relies on the PR algorithm to obtain the final testing results [17]. The realization is more flexible, and it can dynamically test optical elements and systems. It has good application prospects in optical processing, system configuration, active optics, adaptive optics, and other fields, thus favored by experts. The main advantages of the PR measurement method are as follows (see Reference [18] for details): large dynamic range, high resolution, and high sensitivity. It can accurately calculate the surface of the mirror to be measured with small camera sampling points, which can achieve the equivalent accuracy of the interferometer.

In this paper, on the basis of FPWS and algorithm research in the earlier stage [19,20], we will not repeat the detailed research conducted on the FPWS principle, algorithms, and improvement and only simplify and introduce the principle of PR. In order to verify the testing capability of PR technology on complex optical surfaces, an experimental platform for testing different types of surfaces based on modified PR (MPR) was built. In order to verify the feasibility and effectiveness of the MPR method, a large number of repetitive experiments were carried out for verification, and the results of the MPR method were compared with those of the ZYGO interferometer [21,22]. Here, we use a ZYGO interferometer to provide independent metrology of a test optic for direct comparison with the estimates that we obtained with the PR method. The experimental results show that there is a great similarity between the surface error distribution and the peak valley (PV) value and root-mean-square (RMS) value of the error, indicating the feasibility of the FPWS method in the testing of complex optical surfaces.

In this paper, we will first introduce the principle of PR in Section 2. In Section 3, the design of the experiment is presented. The results and discussion are shown in Section 4, and the conclusion is finally drawn in Section 5.

## 2. The Principle of PR

**Figure 1:**
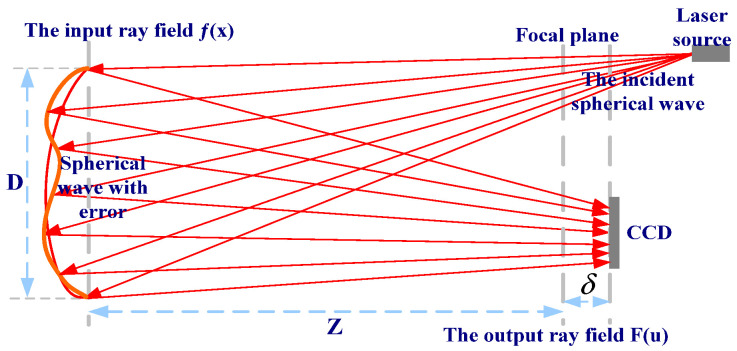
The optical principle of PR.

The PR system is the wave-front detector of focal plane waves. A laser spotlight on the object plane is a target designated from the focal plane image acquisition, using the acquired image, the defocus of the corresponding image, and the known pupil size and shape to reverse solve the aberration of the optical system [23,24]. The structure of the PR system is shown in Figure 1.

**Figure 2:**
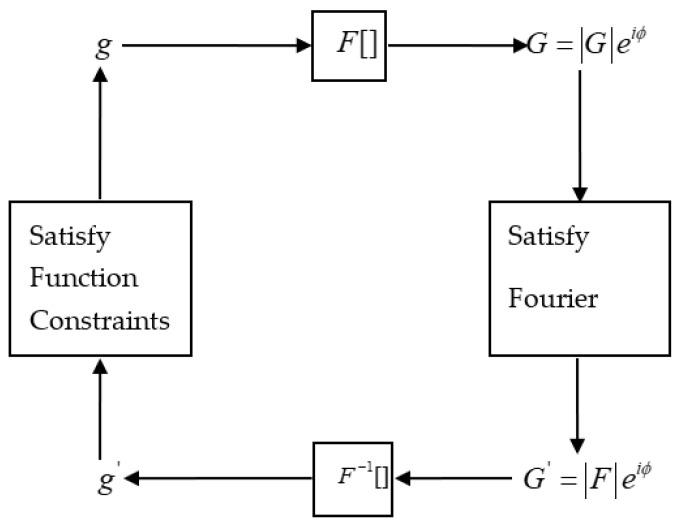
Block diagram of the Gerchberg–Saxton algorithm.

In the optics domain, there is much research on the algorithms of PR, with the core question concerning the Gerchberg–Saxton (GS) algorithm [25]. The GS algorithm was first proposed by Gerchberg et al., as shown in Figure 2, and subsequently appeared in various algorithms [26,27,28,29], such as the gradient search algorithm and input–output algorithm. So, PR technology has been widely used, and the PR algorithm has become the most important research domain because its important applications include wavefront sensing, X-ray crystallography, astronomy, transmission electron microscopy, and coherent diffractive imaging, for which *M* = 2 [30,31,32,33]. Therefore, the PR measurement method has been used by scholars in the research on testing optical freeform surfaces [34,35].

Assuming that the aperture of a measured optical system is *D*, the focal length is *Z*, the center wavelength of the laser source is λ, and its pupil constraint function is |f(x)|, where *x* is a two-dimensional vector, η is a wavefront distortion. Then, for the focal plane, its generalized pupil function is expressed as follows:

(1) f(x)=|f(x)|exp[iη(x)].

Among them, η can be used for fitting via Zernike polynomials: $\eta (x)=\sum_{n}\alpha_{n}Z_{n}(x)$, where real numbers $\alpha_{n}$ represent the *nth* polynomial coefficients, and $Z_{n}$ represent the *nth* Zernike polynomial basis. For linear optical systems, the pulse response function f(x) of the generalized pupil F(u) on the plane with defocus δ is expressed as follows:

(2) $$F(u)=\left|F(u)\right|\exp[i\psi (u)]=F^{-1}\{f(x)\exp[\varepsilon (x,\delta )]\}.$$

Among them, *x* is the pupil domain coordinate, *u* is the image domain coordinate, and *x* and *u* are both two-dimensional vectors, ψ represents the phase part of the pulse response, *F* represents the two-dimensional Fourier transform, $F^{-1}$ represents the two-dimensional inverse Fourier transform, and ε(x,δ) represents the wavefront distortion δ caused by defocusing at position *x*. For a PR system, |f(x)| of Formula (1) is a prior condition for the known optical system under test, corresponding to the size and shape of the pupil. The image |F(u)| is collected via CCD; The defocus amount at the position of the CCD is δ. The purpose of using PR for wavefront sensing is to calculate η(x) based on the above-known quantities.

In our work on the PR algorithm, we mainly pay more attention to the GS algorithm and the gradient search algorithm, which is another common method to solve the PR problem. We have conducted some research on both of the two algorithms. Here, we separately describe them and show the modified PR algorithm and improvement [20].

### 2.1. Gerchberg–Saxton Algorithm

The GS algorithm can be described as follows: the estimated values of $g_{m,k},\theta_{m,k},G_{m,k},\phi_{m,k}$ are for each pair of f,η,F,ψ at the *kth* iteration of the *mth* image; $g_{k}$ represents the joint estimation to f of each pair of $g_{m,k}$ at the *kth* iteration, $g_{k}(x)=\frac{1}{M}\sum_{m=1}^{M}g_{m,k}(x)$. The steps of the GS algorithm are as follows (m∈[1,M]):

Initialization *k* = 0; $\theta_{m,k}$ = 0.

(3) $$a.\;\varepsilon_{m}(x)=\varepsilon (x,\delta_{m})=\frac{\pi \delta_{m}{\left\|x\right\|}^{2}}{\lambda Z^{2}},\;g_{k}(x)=|f(x)|$$

(4) $$b.\;G_{m,k}(u)=\left|G_{m,k}(u)\right|\exp[i\phi_{m,k}(u)]=F\{g_{k}(x)\exp[i\varepsilon_{m}(x)]\}$$

(5) $$c.\;G_{m,k}{}^{\prime }(u)=\left|F(u)\right|\exp[i\phi_{m,k}(u)]$$

(6) $$d.\;g_{m,k}{}^{\prime }(x)=\left|g_{m,k}{}^{\prime }(x)\right|\exp[i\theta_{m,k}{}^{\prime }(u)]=F^{-1}\{G_{m,k}{}^{\prime }(u)\exp[-\varepsilon_{m}(x)]\}$$

(7) $$e.\;g_{m,k+1}(x)=\left|f(x)\right|\exp[i\theta_{m,k+1}(x)]=\left|f(x)\right|\exp[i\theta_{m,k}{}^{\prime }(x)]$$

(8) $$f.\;g_{k+1}(x)=\frac{1}{M}\sum_{m=1}^{M}g_{m,k+1}(x)$$

Repeat b~f until the exit condition is reached, which can be a limit on the number of iterations or a decrease in the objective function to a specified value.

The objective function is expressed as follows:

(9) $$B_{k}=E_{FK}{}^{2}=N^{-2}{\sum_{m=1}^{M}\sum_{u}\left|G_{m,k}(u)-G_{m,k}{}^{\prime }(u)\right|}^{2},$$

where *N* represents the width of the collected image, which is square. According to Formulas (4) and (5), the phase part of $G_{m,k}(u)$ and $G_{m,k}{}^{\prime }(u)$ is equal, so Formula (9) can be transformed into the following:

(10) $$B_{k}=E_{FK}{}^{2}=N^{-2}{\sum_{m=1}^{M}\sum_{u}\left|G_{m,k}(u)-\left|F(u)\right|\right|}^{2}.$$

The entire process of the GS method is shown in Figure 2. As described in Figure 2, the GS algorithm can be applied to problems known to both |F| and |f|. The GS algorithm is actually Newton’s steepest descent method for the objective function (10), so the GS algorithm is convergent.

### 2.2. The Modified PR Algorithm

We apply the mathematical optimization method with Equation (10) as the function of the object and the unknown quantity of each partial derivative together with the substitution gradient search algorithm, finally obtaining the estimation of the wave-front distortion corresponding to θ, when $B_{k}$ is smallest. The most important application of the gradient search algorithm is the correct description of the function of the object and the partial derivatives of each variable. We first discuss the partial derivative g(x), which is the unknown variable. We obtain the derivative from *B* to g(x), respectively, and obtain the derivative from $B_{k}$ to the real part of $\partial g_{real}$ and the imaginary part of $\partial g_{imag}$.

(11) $$\begin{array}{l} \partial g_{real}B_{k}\equiv \frac{\partial B_{k}}{\partial g_{real.k}(x)}=2N^{-2}\sum_{m=1}^{M}\sum_{u}\left[\left|G_{m,k}(u)\right|-\left|F(u)\right|\right]\frac{\partial \left|G_{m,k}(u)\right|}{\partial g_{real,k}(x)}\\ \partial g_{imag}B_{k}\equiv \frac{\partial B_{k}}{\partial g_{imag.k}(x)}=-i2N^{-2}\sum_{m=1}^{M}\sum_{u}\left[\left|G_{m,k}(u)\right|-\left|F(u)\right|\right]\frac{\partial \left|G_{m,k}(u)\right|}{\partial g_{imag,k}(x)} \end{array},$$

where

(12) $$\begin{array}{l} \frac{\partial \left|G_{m,k}(u)\right|}{\partial g_{real,k}(x)}=\frac{\partial }{\partial g_{real,k}(x)}\sum_{y}g_{k}(y)\exp[i\varepsilon_{m}(x)]\exp[\frac{-i2\pi uy}{N}]=\exp[i\varepsilon_{m}(x)]\exp[\frac{-i2\pi ux}{N}]\\ \frac{\partial \left|G_{m,k}(u)\right|}{\partial g_{imag,k}(x)}=\frac{\partial }{\partial g_{imag,k}(x)}\sum_{y}g_{k}(y)\exp[i\varepsilon_{m}(x)]\exp[\frac{-i2\pi uy}{N}]=\exp[i\varepsilon_{m}(x)]\exp[\frac{-i2\pi ux}{N}] \end{array},$$

and

(13) $$\begin{array}{l} \frac{\partial \left|G_{m,k}(u)\right|}{\partial g_{real,k}(x)}=\frac{\partial {\left[{\left|G_{m,k}(u)\right|}^{2}\right]}^{1/2}}{\partial g_{real,k}(x)}=\frac{1}{2\left|G_{m,k}(u)\right|}\frac{\partial {\left|G_{m,k}(u)\right|}^{2}}{\partial g_{real,k}(x)}=\frac{G(u)\exp[-i\varepsilon_{m}(x)+i2\pi ux/N]}{2\left|G(u)\right|}+c.c.\\ \frac{\partial \left|G_{m,k}(u)\right|}{\partial g_{imag,k}(x)}=\frac{\partial {\left[{\left|G_{m,k}(u)\right|}^{2}\right]}^{1/2}}{\partial g_{imag,k}(x)}=\frac{1}{2\left|G_{m,k}(u)\right|}\frac{\partial {\left|G_{m,k}(u)\right|}^{2}}{\partial g_{imag,k}(x)}=\frac{G(u)\exp[-i\varepsilon_{m}(x)+i2\pi ux/N]}{2\left|G(u)\right|}+c.c. \end{array}.$$

Thus, Equation (11) can be changed to the following:

(14) $$\begin{array}{l} \partial g_{real}B_{k}=N^{-2}\sum_{m=1}^{M}\sum_{u}\left[G_{m,k}(u)-\left|F(u)\right|G_{m,k}(u)/\left|G_{m,k}(u)\right|\right]=\frac{-iG(u)\exp[-i\varepsilon_{m}(x)+i2\pi ux/N]}{2\left|G(u)\right|}+c.c.\\ \partial g_{imag}B_{k}=-iN^{-2}\sum_{m=1}^{M}\sum_{u}\left[G_{m,k}(u)-\left|F(u)\right|G_{m,k}(u)/\left|G_{m,k}(u)\right|\right]=\frac{-iG(u)\exp[-i\varepsilon_{m}(x)+i2\pi ux/N]}{2\left|G(u)\right|}+c.c. \end{array},$$

where c.c. represents the former plural conjugate.

Using $G_{m,k}{}^{\prime }(u)=\left|F(u)\right|\exp[i\phi_{m,k}(u)],m\in [1,M]$ (Equation (5)) to define $G_{m,k}{}^{\prime }(u)$, we can obtain $G_{m,k}{}^{\prime }(u)=\left|F(u)\right|G_{m,k}(u)/\left|G_{m,k}(u)\right|$.

Thus, Equation (14) can be expressed as follows:

(15) $$\begin{array}{l} \partial g_{real}B_{k}=2Real\sum_{m}\left[g_{m,k}(x)-g_{m,k}{}^{\prime }(x)\right]\\ \partial g_{imag}B_{k}=2Imag\sum_{m}\left[g_{m,k}(x)-g_{m,k}{}^{\prime }(x)\right] \end{array}.$$

We consider θ(x) as the derivative of the unknown value. From Equation (3), we obtain the derivative from $B_{k}$ to θ(x) as follows:

(16) $$\partial_{\theta }B_{k}=\frac{\partial B_{k}}{\partial \theta_{k}(x)}=2N^{-2}\sum_{m}\sum_{u}\left[\left|G_{m,k}(u)\right|-\left|F(u)\right|\right]\frac{\partial \left|G_{m,k}(u)\right|}{\partial \theta_{k}(x)}.$$

Because of

(17) $$\frac{\partial \left|G_{m,k}(u)\right|}{\partial \theta_{k}(x)}=\frac{\partial }{\partial \theta_{k}(x)}\sum_{y}\left|f(y)\right|\exp[i\theta (y)]\exp[i\varepsilon_{m}(x)]\exp[\frac{-i2\pi uy}{N}]=ig_{k}(x)\exp[i\varepsilon_{m}(x)]\exp[\frac{-i2\pi ux}{N}],$$

we can obtain the following:

$$\frac{\partial \left|G_{m,k}(u)\right|}{\partial \theta_{k}(x)}=\frac{G_{m,k}(u)(-i)g_{k}^{*}(x)\exp[-i\varepsilon_{m}(x)]\exp[i2\pi ux/N]+c.c.}{2\left|G_{m,k}(u)\right|}.$$

Thus, we can obtain the following:

(18) $$\begin{array}{ll} \partial_{\theta }B_{k} & =\sum_{m}ig_{m,k}{}^{*}(x)[g_{m,k}{}^{\prime }(x)-g_{m,k}(x)]+c.c.\\ \; & \underset{}{}\underset{}{}{\underset{}{}}^{}=-2Imag\sum_{m}\left[g_{m,k}{}^{*}(x)g_{m,k}{}^{\prime }(x)\right]\\ \; & \underset{}{}\underset{}{}{\underset{}{}}^{}=-2\left|f(x)\right|\sum_{m}\left|g_{m,k}{}^{\prime }(x)\right|\sin[\theta_{m,k}{}^{\prime }(x)-\theta_{m,k}(x)] \end{array}.$$

We consider the Zernike coefficient α(x) as the derivation of the unknown value. From Equation (10), we obtain the derivative from $B_{k}$ to α(x) as follows:

(19) $$\frac{\partial B_{k}}{\partial \alpha_{n,k}}=\sum_{x}\frac{\partial B}{\partial \theta_{k}(x)}\frac{\partial \theta_{k}(x)}{\partial \alpha_{n,k}(x)}$$

Take $\frac{\partial \theta_{k}(x)}{\partial \alpha_{n,k}(x)}=\frac{\partial }{\partial \alpha_{n,k}}[\sum_{n=1}^{m}\alpha_{n,k}Z_{n}(x)]=Z_{n}(x)$ into Equation (19). We obtain the objective function, which is calculated as follows:

(20) $$\partial_{\alpha_{n}}B_{k}=-2\sum_{m}\sum_{x}\left|f(x)\right|\left|g_{m,k}{}^{\prime }(x)\right|\sin[\theta_{m,k}{}^{\prime }(x)-\theta_{m,k}(x)]Z_{n}(x).$$

With the objective Equation (10) and its impact on the Zernike coefficient derivative Equation (20), we can use the mathematical optimization algorithm, such as the Limited-memory BFGS algorithm, to solve various Zernike wave-front coefficient values.

The GS method is equivalent to Newton’s steepest descent method with (10) as the objective function. To simplify the problem, we set *M* = 1, and Equation (15) can be expressed as $\partial_{g}B=2\left[g(x)-g^{\prime }(x)\right]$. The step size along the gradient can be determined via the first term of the Taylor series expansion of *B*, which is expressed as follows: $B\approx B_{k}+\sum_{x}\partial_{g}B_{k}\left[g(x)-g_{k}(x)\right]$. When $g(x)=g_{k}{}^{\prime \prime }(x)$, the first term of the expansion term *B* is zero; thus, $g_{k}{}^{\prime \prime }(x)-g_{k}(x)=-B_{k}\partial_{g}B_{k}/\sum_{y}{\left(\partial_{g}B_{k}\right)}^{2}$ and $g_{k}{}^{\prime \prime }(x)-g_{k}(x)=-(1/4)\partial_{g}B_{k}=(1/2)\left[g_{k}^{\prime }(x)-g_{k}(x)\right]$. So, the GS method is equivalent to Newton’s steepest descent method with B as the objective function, with a step size of $(1/2)\left[g_{k}^{\prime }(x)-g_{k}(x)\right]$. We can predict that for the same target wavefront, the GS algorithm and MPR algorithm will be applied separately. At the beginning of the iteration, the convergence speed of the GS algorithm will be slightly faster than the modified PR algorithm, but in the subsequent iteration process, the convergence speed of the GS algorithm will be significantly slower than the modified PR algorithm. This is consistent with the phenomenon that Newton’s steepest direction descent method and conjugate gradient method are used for the same problem in optimization problems. Thus, we know that our algorithm is convergent.

In order to verify the ability to test the complex mirror with the MPR method and the equivalent accuracy to that of the ZYGO interferometer. In this paper, we will elucidate the design of the experiments and perform some comparable experiments with MPR and ZYGO interferometers, which aim to demonstrate the measurement ability and equivalent accuracy to that of the interferometer in the complex surface testing with the MPR method.

## 3. The Design of the Experiments

### 3.1. Measuring Plane Mirror

**Figure 3:**
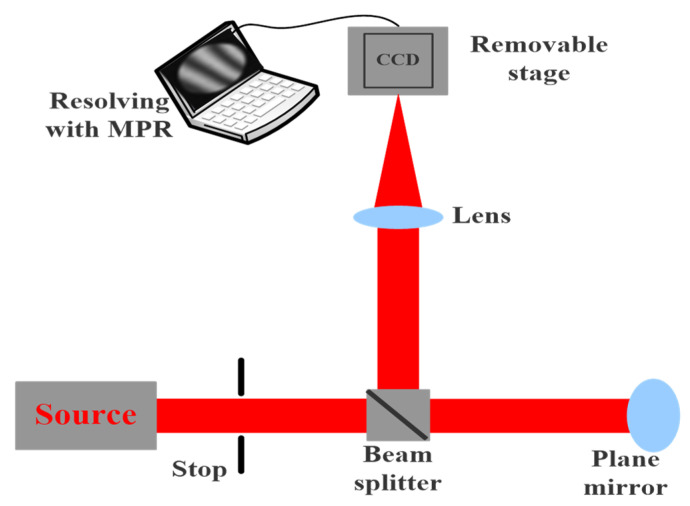
The optical path structure of the MPR measurement method.

**Figure 4:**
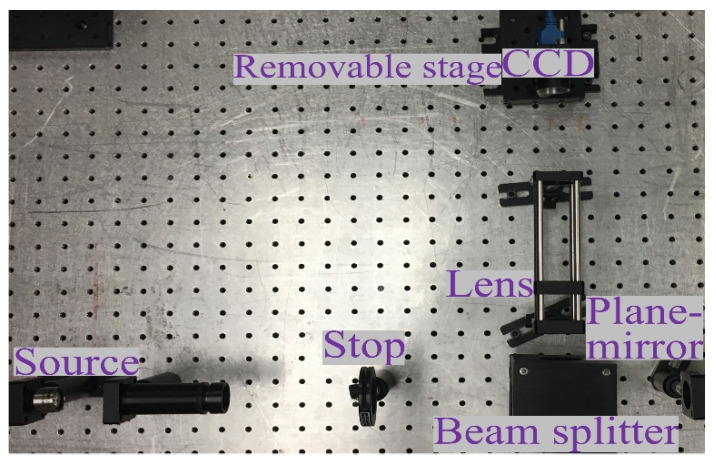
The setup experiment chart with MPR on plane mirror.

Before starting the measurement experiment, in order to obtain accurate results, we first measure the plane mirror with a good surface shape to determine the system error. The optical path structure of the MPR measurement method is shown in Figure 3, and the experimental diagram is shown in Figure 4. The wavelength is 632.8 nm, the diaphragm is 10 mm, the lens focal length is 150 mm, the central exit pupil aperture is 10 mm, and the size of the plane mirror is 25.4 mm. The defocusing amount is selected in the experiment. The camera pixel size is 6.45 μm. Each defocusing position intercepts the pixel size area centered on the target, the exposure time is 20 ms, the camera bottom is placed on the electric translation platform, and the accuracy of the mobile platform is ±5 μm.

**Figure 5:**
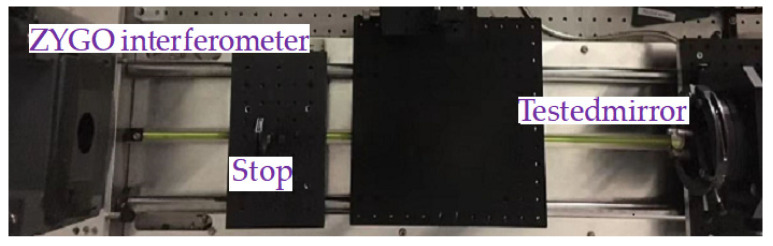
Testing mirror with ZYGO interferometer.

After adjusting the optical path accurately, the MPR measurement method and ZYGO interferometer are used to measure the plane mirror, respectively. The testing with the ZYGO interferometer is shown in Figure 5. Here, we briefly describe how we use the ZYGO interferometer to test the shape of the surface to be measured. Firstly, we use a standard plane mirror for calibration. The cross and interference fringes of the interferometer were adjusted to minimize the wavefront difference. When measuring spherical mirrors, such as the testing of convex and concave mirrors, it is related to the number of F. In general, if the interferometer reference environment *F* is small, the testing environment *F* is large (the number of reference environment *F* is smaller than the number of *F* to be detected). The radius of the mirror to be measured is smaller than the moving length (the moving range of the interferometer). The spherical wave of the convex lens parallel light reference mirror is vertically incident on the convex lens. Then, the reflected light returns to the spherical wave in an original way. After passing through the reference mirror, it becomes a plane wavefront (horizontal light). The parallel light passes through the reference mirror and becomes a spherical wavefront. The spherical wavefront is vertically incident on the last surface of the reference mirror, causing two types of reflection and transmission. The reference light transmitted by the reflected light continues to propagate until it is vertically incident on the test mirror; then, it is reflected back by the test mirror. The original path returns to the formed test light, and the reference light interferes with the test light, forming interference fringes. By using the software provided by the interferometer, edge areas are removed, reasonable measurement modes are selected, and reasonable data fitting methods are selected during the fitting process.

**Figure 6:**
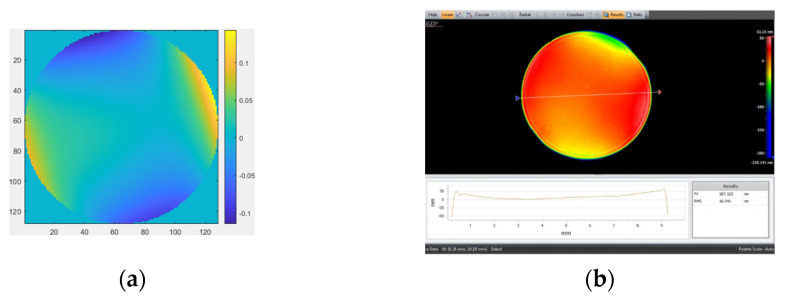
The measurement results by removing the first four items. (**a**) MPR: RMS = 0.0475 λ, PV = 5.3352 λ; (**b**) ZYGO interferometer: RMS = 0.037 λ, PV = 4.149 λ.

**Figure 7:**
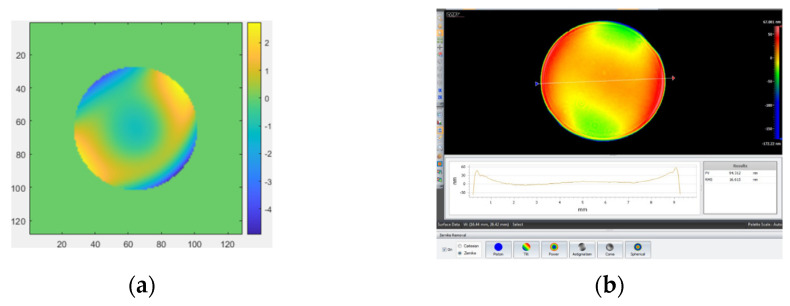
The measurement results by removing the first four items and spherical. (**a**) MPR: RMS = 0.04392 λ, PV = 4.9304 λ; (**b**) ZYGO interferometer: RMS = 0.03325 λ, PV = 3.7685 λ.

The measurement results obtained by removing the first four items (piston, tilt X, tilt Y, and power) with MPR are shown in Figure 6a. The measurement result obtained by removing the first four items (piston, tilt X, tilt Y, and power) with the ZYGO interferometer is shown in Figure 6b. The measurement result obtained by removing the first four items (piston, tilt X, tilt Y, power, and spherical) with MPR is shown in Figure 7a. The measurement results obtained by removing the first four items (piston, tilt X, tilt Y, power, and spherical aberration) with the ZYGO interferometer are shown in Figure 7b. In order to obtain accurate measurements, the aberration of the system itself should be subtracted whenever the complex mirror is measured.

In order to compare the experimental results more effectively, we need to subtract the systematic error (the mean results of Figure 6 and Figure 7) in the following measurement experiments, in which each measurement results minus the systematic error equals the final measurement results.

### 3.2. Measuring Concave Mirror

**Figure 8:**
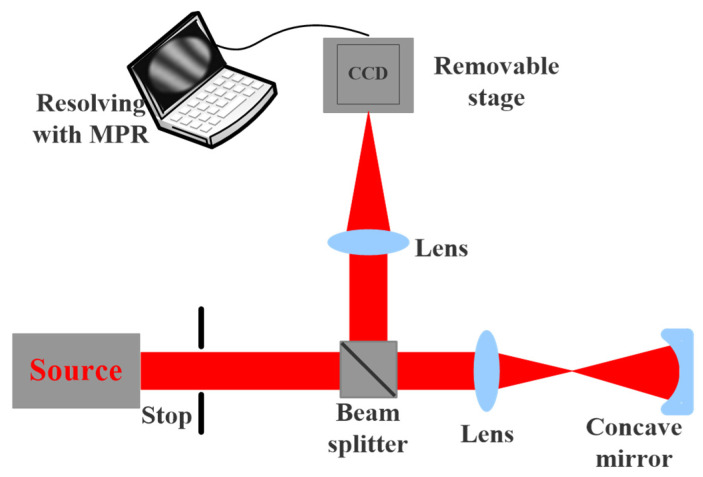
The structure chart of the MPR light path.

**Figure 9:**
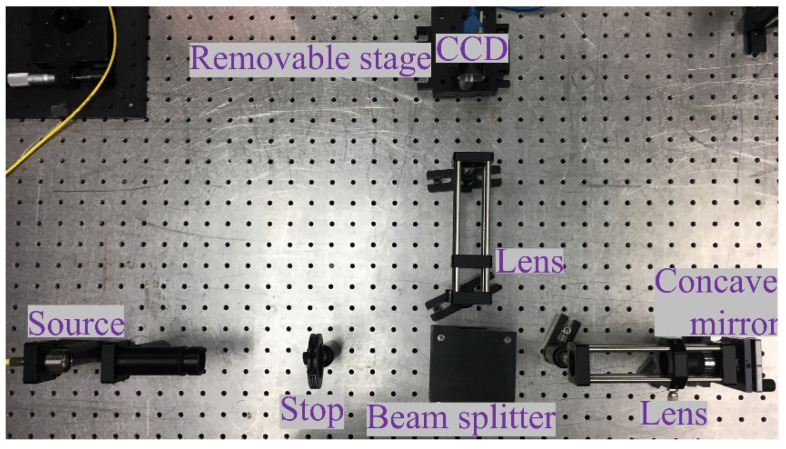
The setup experiment chart with MPR.

**Figure 10:**
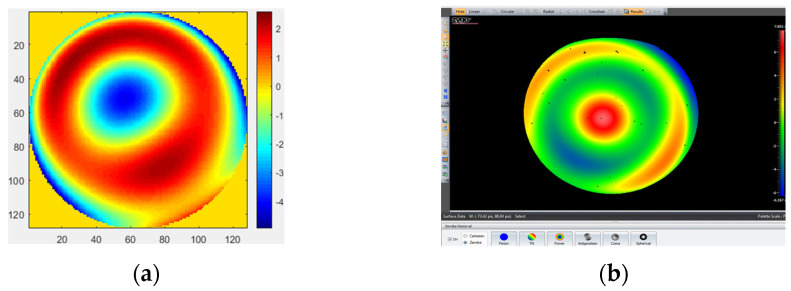
The measurement results. (**a**) MPR: RMS = 2.5322 λ, PV = 15.529 λ; (**b**) ZYGO interferometer: RMS = 2.5139 λ, PV = 14.119 λ.

The size of the concave mirror (in order to make a clear distinction, we call the concave mirror the spherical concave mirror I) to be measured is 25.4 mm. The optical structure of the MPR measurement method is shown in Figure 8, and the experimental diagram is shown in Figure 9. The MPR measurement method and ZYGO interferometer are used to measure the plane mirror. The measurement results obtained by removing the first four items (piston, tilt X, tilt Y, and power) are shown in Figure 10.

### 3.3. Measuring Spherical Mirror

**Figure 11:**
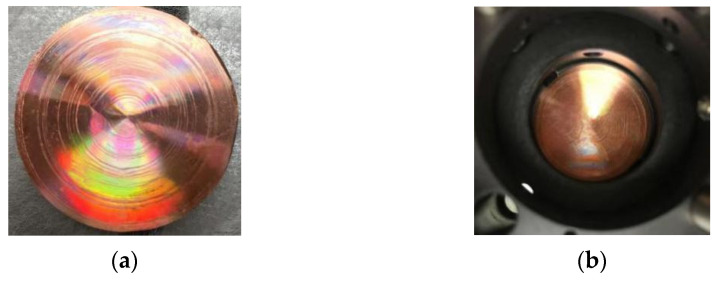
The tested mirrors: (**a**) spherical concave mirror II and (**b**) aspherical mirror.

**Figure 12:**
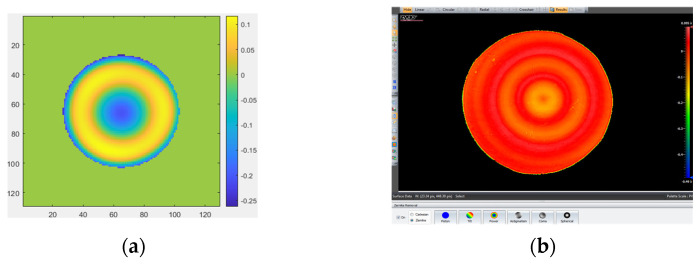
The measurement results obtained by removing the first four items (piston, tilt X, tilt Y, and power) on spherical mirror II. (**a**) MPR: RMS = 0.0527 λ, PV = 0.667 λ; (**b**) ZYGO interferometer: RMS = 0.041 λ, PV = 0.585 λ.

**Figure 13:**
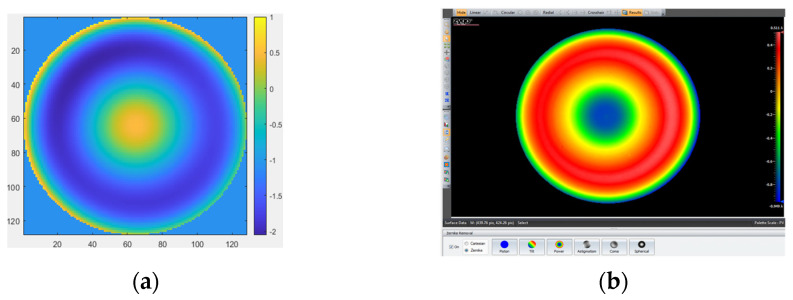
The measurement results of aspherical mirror. (**a**) MPR: RMS = 0.667 λ, PV = 3.027 λ; (**b**) ZYGO interferometer: RMS = 0.632 λ, PV = 2.155 λ.

The size of the spherical mirror (here, it is spherical concave mirror II) to be measured is 25.4 mm, as shown in Figure 11, (a) for spherical mirror II and (b) for the aspherical mirror. The MPR measurement method and ZYGO interferometer are, respectively, used to measure the spherical mirror II and aspherical mirror. The measurement results obtained by removing the first four items (piston, tilt X, tilt Y, and power) on spherical mirror II are shown in Figure 12, and the aspherical mirror measurement results obtained are shown in Figure 13.

We can see that although the topological geometry and PV/RMS of both MPR and ZYGO interferometer are the same, there is a little difference in the RMS value from the above measurement results The difference in PV is relatively large, partially due to the following reasons. Firstly, there is a smoothing process when using the ZYGO interferometer, which the solution process of the MPR method does not have. Secondly, during the solution process, we calculated the whole mask circular area with the MPR but measured results via the ZYGO interferometer after removing boundary Burr. Therefore, although the RMS of the whole mask cannot be greatly affected, it will be greatly different from PV. The experimental results show that there is a great similarity between the surface error distribution, PV value, and RMS value of the error, indicating the feasibility of the FPWS method in testing complex optical surfaces.

## 4. Results and Discussion

The comparison results of measurement experiments are shown in Table 1. The measurement results with wavefront-sensing technology based on image information of the focal plane were compared with the measurement results with the ZYGO interferometer. The experimental results demonstrate that good agreement is obtained among the errors distribution, PV value, and RMS value of the ZYGO interferometer, which shows the feasibility and validity of wavefront sensing based on image information of focal plane technology in optical metrology for the complex optical surface.

Above all the experimental results, we can not only easily see that MPR technology has the testing capability on complex optical surfaces with an experimental platform for testing different types of surfaces but also explain that the MPR method has the feasibility and effectiveness in a large number of repetitive experiments by comparing the results of MPR method with those of the ZYGO interferometer. Furthermore, the MPR method has the following advantages that a ZYGO interferometer does not have: (1) The impact of platform vibration on the PR system is small, even negligible; (2) PR system has a simple structure and can even detect the whole optical system in place via the existing camera on the imaging system without any change to the optical path; (3) Better measurement accuracy can be obtained with fewer sampling points by CCD in the PR system. From the above results by comparing the MPR method and ZYGO interferometer, we not only proved the ability to test complex optical surfaces but also showed that the MPR method had advantages that the ZYGO interferometer did not have; thus, we could use the MPR method to institute the ZYGO interferometer in the bad environment, especially vibrations and disturbances. For example, phasing the James Webb Space Telescope just used the PR method not interferometer in order to correct the deployment errors and produce diffraction-limited images, wavefront sensing, and controls process was executed to adjust each of the optical elements of JWST.

## 5. Conclusions

In this paper, comparing the results of the MPR with the results of the ZYGO interferometer shows that the proposed MPR method is feasible for measuring complex surfaces. The difference in PV is relatively large, partially due to the following reasons: there is a smoothing process when using the ZYGO interferometer, which the solution process of the modified PR method does not have; during the solution process, we calculated the whole mask circular area with the MPR but measured results via the ZYGO interferometer after removing boundary Burr. Therefore, although the RMS of the whole mask cannot be greatly affected, it will be greatly different from PV. The experimental results show that there is a great similarity between the surface error distribution, PV value, and RMS value of the error, indicating the feasibility of the FPWS method in the testing of complex optical surfaces. Based on the research progress at home and abroad, the testing methods and key problems in the measurement of complex optical surfaces are analyzed and studied. As far as the testing methods of complex optical surfaces are concerned, non-contact measurement has become an important developmental direction with its own advantages. It has guiding significance for our future research on large-aperture complex optical surface testing technology. At present, the following testing methods of complex optical surfaces are combined: small size and high precision, on-line measurement, full-band high-precision measurement, large dynamic range, simple structure, and low cost. All these superiorities of this testing method have important practical significance and broad application prospects.

## Figures and Tables

**Table 1:** The comparison results of measurement experiments.

The Type of Tested Mirror	Evaluation Index	MPR	ZYGO	RMSD ifference
Plane mirror (removing the first four items)	RMS PV	0.0475 λ 5.3352 λ	0.037 λ 4.149 λ	0.0105 λ
Plane mirror (removing the first four items and spherical aberration)	RMS PV	0.04392 λ 4.9304 λ	0.03325 λ 3.7685 λ	0.01067 λ
Spherical concave mirror I	RMS PV	2.5322 λ 15.529 λ	2.5139 λ 14.119 λ	0.0183λ
Spherical concave mirror II	RMS PV	0.0527 λ 0.667 λ	0.041 λ 0.585 λ	0.0117 λ
Aspherical mirror	RMS PV	0.667 λ 3.027 λ	0.632 λ 2.155 λ	0.035 λ
